# Designing and making device and information system of road accidents in order to reduce the mortality rate due to brain deaths

**Published:** 2012-11

**Authors:** Alireza Saleki, Feizollah Foroughi, Hanieh Shahandeh, Koorosh Hamzehee, Touraj Shirzadian, Mohammad Mahbobi, Parviz Ghaffari, Mohammad Salimi

**Affiliations:** ^*a*^Social Development Research Center, Vice Chancellory for Research and Technology, Kermanshah University of Medical Sciences, Kermanshah, Iran.; ^*b*^Scientometrics Unit , Vice Chancellory for Research and Technology, Kermanshah University of Medical Sciences, Kermanshah, Iran.; ^*c*^Evaluation Unit , Vice Chancellory for Research and Technology, Kermanshah University of Medical Sciences, Kermanshah, Iran.; ^*d*^Research Projects Unit , Vice Chancellory for Research and Technology, Kermanshah University of Medical Sciences, Kermanshah, Iran.; ^*e*^Industrial Relation Uint , Vice Chancellory for Research and Technology, Kermanshah University of Medical Sciences, Kermanshah, Iran.; ^*f*^Departmen Orthoped Surgery , Sch. Med, Kermanshah University of Medical Sciences, Kermanshah, Iran.

## Abstract

**Background:**

In road accidents, most of the deaths occur in the absence of medical cares. Accident severity index (ASI) measures the seriousness of an accident. It is defined as the number of persons killed per 100 accidents. According to world statistics global average value of ASI is one, whereas in Iran is 10. Also, one brain death occurs per 100 accidents. Although some portions of these mortalities are inevitable due to the initial injuries, a significant number of injuries that lead to death can be prevented.

Therefore, the aim of this study was to developing and implementing a wise system to automatically inform the authorized organizations of the road accident as well as the patients’ vital signs and geographical location of accident in the shortest possible time.

**Methods:**

In this study, at first the flowchart was designed schematically, then, executive processes were performed. In first step, the hardware schematic of inside of the car was designed which was followed by programming the software for communication and data delivery was done.

**Results:**

After installing the hardware and communicating system, when the trauma sensor was triggered, an electrode appeared and a massage with the title "If the driver can continue his trip or not " was broadcasted immediately. If the driver or passengers can press the interrupting massage button , it means that he is physically healthy so he can continue the trip, otherwise, the global positioning system (GPS) devise activates at a specific time which is determined by a delay maker and the first short message (SMS) is sent through the General Packet Radio Service (GPRS) system to the information receiving center (a hypothetical medical emergency center) and following a vocal alarm in an emergency center, the exact geographical location of the accident is defined and in the monitoring system became invisible.

**Conclusions:**

This system is made on trial and error basis, thus it is necessary to place in an evolutionary algorithm like the other technological products so that it will be developed professionally under a systematic and expertized manufacturing plan.

**Keywords:**

Road accidents, Mortality, Information system, Brain deaths


					Volume 4, Suppl. 1 Nov 2012
Publisher: Kermanshah University of Medical Sciences
URL: http://www.jivresearch.org
Copyright:
Congress of Iranian Neurosurgeons, 14-16 November 2012, Kermanshah, Iran
Paper No. 88
Designing and making device and information system of road
accidents in order to reduce the mortality rate due to brain
deaths
Alireza Saleki a
, Feizollah Foroughi b,
*, Hanieh Shahandeh c
, Koorosh Hamzehee d
, Touraj Shirzadian e
,
Mohammad Mahbobi d
, Parviz Ghaffari f
, Mohammad Salimi d
a
Social Development Research Center, Vice Chancellory for Research and Technology, Kermanshah University of Medical
Sciences, Kermanshah, Iran.
b
Scientometrics Unit , Vice Chancellory for Research and Technology, Kermanshah University of Medical Sciences,
Kermanshah, Iran.
c
Evaluation Unit , Vice Chancellory for Research and Technology, Kermanshah University of Medical Sciences, Kermanshah,
Iran.
d
Research Projects Unit , Vice Chancellory for Research and Technology, Kermanshah University of Medical Sciences,
Kermanshah, Iran.
e
Industrial Relation Uint , Vice Chancellory for Research and Technology, Kermanshah University of Medical Sciences,
Kermanshah, Iran.
f
Departmen Orthoped Surgery , Sch. Med, Kermanshah University of Medical Sciences, Kermanshah, Iran.
Abstract:
Background: In road accidents, most of the deaths occur in the absence of medical cares. Accident severity index
(ASI) measures the seriousness of an accident. It is defined as the number of persons killed per 100 accidents.
According to world statistics global average value of ASI is one, whereas in Iran is 10. Also, one brain death occurs
per 100 accidents. Although some portions of these mortalities are inevitable due to the initial injuries, a significant
number of injuries that lead to death can be prevented.
Therefore, the aim of this study was to developing and implementing a wise system to automatically inform the
authorized organizations of the road accident as well as the patients' vital signs and geographical location of
accident in the shortest possible time.
Methods: In this study, at first the flowchart was designed schematically, then, executive processes were performed.
In first step, the hardware schematic of inside of the car was designed which was followed by programming the
software for communication and data delivery was done.
Results: After installing the hardware and communicating system, when the trauma sensor was triggered, an
electrode appeared and a massage with the title "If the driver can continue his trip or not " was broadcasted
immediately. If the driver or passengers can press the interrupting massage button , it means that he is physically
healthy so he can continue the trip, otherwise, the global positioning system (GPS) devise activates at a specific time
which is determined by a delay maker and the first short message (SMS) is sent through the General Packet Radio
Service (GPRS) system to the information receiving center (a hypothetical medical emergency center) and following a
vocal alarm in an emergency center, the exact geographical location of the accident is defined and in the monitoring
system became invisible.
Conclusions: This system is made on trial and error basis, thus it is necessary to place in an evolutionary algorithm
like the other technological products so that it will be developed professionally under a systematic and expertized
manufacturing plan.
Keywords:
Road accidents, Mortality, Information system, Brain deaths
* Corresponding Author at:
Feizollah Foroughi: Scientometrics Unit, Vice Chancellory for Research and Technology , Kermanshah University of Medical University, Kermanshah,
Iran. Tel: +988318360014, Email: fforoughi@yahoo.com, (Foroughi F.).

				

